# Beyond PD-L1 Markers for Lung Cancer Immunotherapy

**DOI:** 10.3390/ijms20081915

**Published:** 2019-04-18

**Authors:** Kamila Wojas-Krawczyk, Ewa Kalinka, Anna Grenda, Paweł Krawczyk, Janusz Milanowski

**Affiliations:** 1 Department of Pneumonology, Oncology and Allergology, Medical University of Lublin, 20-090 Lublin, Poland; kamilawojas@wp.pl (K.W.-K.); krapa@poczta.onet.pl (P.K.); jmilanowski@op.pl (J.M.); 2 Clinical Oncology Unit, Clinic of Clinical Oncology and Breast Diseases Polish Mother’s Memorial Hospital, Research Institute, 93-338 Łódź, Poland; ewakalinka@wp.pl

**Keywords:** NSCLC, immune-check points inhibitors, PD-1, PD-L1, tumor mutation burden, tumor immunophenotype, microbiome

## Abstract

Immunotherapy using immune checkpoints inhibitors has become the standard treatment for first and second line therapy in patients with non-small cell lung cancer (NSCLC). However, proper predictive factors allowing precise qualification of NSCLC patients for immunotherapy have not been developed so far. Expression of PD-L1 on tumor cells and tumor mutation burden are used in qualification of patients to first line therapy with pembrolizumab and atezolizumab in combination with ipilimumab in prospective clinical trials. Nevertheless, not all patients with these predictive factors benefit from immunotherapy. Major methodological difficulties in testing of these factors and in the interpretation of test results still exist. Therefore, other predictive factors are sought. Intensive research on the recognition of tumor immunophenotype and gut microbiome in NSCLC patients are underway. The first correlations between the effectiveness of immunotherapy and the intensity of inflammatory response in the tumor, microbiome diversity, and the occurrence of certain bacterial species in gut have been described. The purpose of our manuscript is to draw attention to factors affecting the efficacy of immunotherapy with anti-PD-L1 antibodies in NSCLC patients. Additional markers, for example TMB (tumor mutations burden) or microbiome profile, are needed to more accurately determine which patients will benefit from immunotherapy treatment.

## 1. Introduction

The success of immunotherapy with immune checkpoints inhibitors (ICIs) significantly expanded the horizons of treatment options in non-small cell lung cancer (NSCLC) patients. Blockade of the PD-1 (Programmed Death 1) on lymphocytes or PD-L1 (Programmed Death Ligand 1) on tumor cells, or on infiltrating immune cells, activates effector T cells and their anti-tumor activity. Activation of anti-tumor immune response has been shown to be an effective treatment method in some NSCLC patients. Currently, four immunotherapeutics are registered in European Union and in USA for the therapy in NSCLC patients. Nivolumab (anti-PD-1 monoclonal antibody) has been approved for the second line treatment in patients with locally advanced and advanced NSCLC, and for the first line treatment in combination with ipilimumab in metastatic NSCLC patients with high tumor mutation burden (TMB). Pembrolizumab (another anti-PD-1 monoclonal antibody) is registered for the second line treatment in locally advanced and advanced NSCLC patients with PD-L1 expression on ≥1% of tumor cells, and for the first line treatment in metastatic NSCLC patients with PD-L1 expression on ≥50% of cancer cells. Pembrolizumab in combination with the first line chemotherapy is also indicated in advanced NSCLC patients regardless of PD-L1 expression on tumor cells. Atezolizumab (the first anti-PD-L1 antibody) has been approved for NSCLC patients in the first and in the second line treatment regardless of PD-L1 expression on tumor cells. Patients with metastatic NSCLC may benefit from the first line combination therapy with atezolizumab, bevacizumab and chemotherapy, while patients with locally advanced and metastatic NSCLC from second line atezoliuzmab monotherapy. Durvalumab (anti-PD-L1 antibody) is effective in maintenance therapy in stage IIIA NSCLC patients with PD-L1 expression on ≥1% of tumor cells and without progression after effective concurrent chemoradiotherapy. The registration of durvalumab in this indication has been performed in the European Union and in the USA. Moreover, efficacy of durvalumab plus tremelimumab and durvalumab monotherapy was examined in third line therapy in NSCLC patients (ARCTIC study). Combination of durvalumab and tremelimumab in patients with low or negative expression of PD-L1 on tumor cells did not improve progression free survival and overall survival compared to standard-of-care chemotherapy. Durvalumab monotherapy showed a clinically meaningful reduction in the risk of death compared to chemotherapy in patients with PD-L1 expression on ≥25% of tumor cells. In JAVELIN Lung 200 clinical trial, the efficacy and safety of avelumab (new anti-PD-L1 antibody) were investigated in second line treatment of NSCLC patients. Compared with docetaxel, avelumab did not improve overall survival in patients with PD-L1-positive NSCLC previously treated with platinum-based chemotherapy [1,2,3,4,5,6,7,8,9,10,11,12,13,14].

Although immunotherapy can be effective in NSCLC treatment in some patients, providing durable remissions, it can also develop several immune-related adverse events due to over-stimulation of immune system. Monoclonal antibodies anti-PD-1 or anti-PD-L1 are generally less toxic than chemotherapy. Nevertheless, they can cause mild or serious toxicity mainly affecting the endocrine organs or mucosa of the large intestine and bronchi. Serious side effects of immunotherapy include immune-related colitis, hepatitis, intestinal pneumonitis, thyroiditis, hypophysitis and others. Occurrence of particular stage of toxicities according to Common Toxicity Criteria (CTC) acquires appropriate procedures. Immunotherapy should be continued with close monitoring for grade 1 toxicities, with the exception of some neurologic, hematologic, pulmonary and cardiac toxicities. Immunotherapy may be suspended for most grade 2 toxicities, with consideration of resuming when symptoms revert to grade 1 or less. Corticosteroids may be administered. Appearance of severe toxicities is an indication for discontinuation of immunotherapy. However, after the reduction of side effects, it is possible to return to immunotherapy. Grade 3 toxicities generally warrant suspension of immunotherapy and the initiation of high-dose corticosteroids for at least 4–6 weeks. In general, permanent discontinuation of immunotherapy is recommended with grade 4 toxicities, with the exception of endocrinopathies that have been controlled by hormone replacement. Serious interstitial pneumonia and hepatitis in grade 3 or 4 are an indication for the complete discontinuation of immunotherapy. In less than 10% of patients, immunotherapy should be permanently discontinued because of serious adverse events [15].

Moreover, immunotherapy may accelerate tumor progression in a significant subset of patients ranging from 4% to 30%. The definition of hyperprogression is not fully established. Hyperprogression confirmed in the first imaging examination after immunotherapy start is associated with older age, higher metastatic load, previous irradiation, and *EGFR* gene mutations presence. Hyperprogression should be distinguished from the pseudoprogression associated with increased infiltration of tumor by immune cells [16].

Many unknowns remain to be explained in immunotherapy of cancer patients. One of them is the difficulty in patients’ qualification to immunotherapy based on predictive factors.

Therapeutic indications and predictive factors for immunotherapy with anti-PD-1 and anti-PD-L1 antibodies in NSCLC patients are very diverse. Expression of PD-L1 on tumor cells and TMB are neither the only nor the perfect predictors for immunotherapy. 

## 2. Theory of Immune-Check Points

The cancer immunoediting phenomenon is defined by three stages: elimination, equilibrium, and escape. In the elimination stage, immunosurveillance leads to tumor elimination by proper priming and effector phase of the host immune response. In the equilibrium phase, the immune system does not fully control the malignant cells but despite that it can control the malignancy by inhibiting cancer progression. In the escape phase, the immune system does not control the malignancy, passively allowing proliferation and tumor growth [17]. Thus, the ideal therapeutic intervention would lead from immune escape to elimination phase. Strategies allowing achievement of equilibrium phase are not curative, but possibly lead to overall survival (OS) improvement despite the lack of cancer elimination. As NSCLC cells are moderately immunogenic, equilibrium seems a promising and realistic goal for immune checkpoint inhibitors.

T lymphocyte activation and cellular response occur through a complex interaction between antigen-presenting cell (APC) and T cell. Recognition of antigens on MHC (Major Histocompatibility Complex) molecule by T cell receptor (TCR) is not enough for immune response development. A second signal provided by members of the B7 family on APC is required. CD28 is the primary co-stimulatory signal for the activation of T cells after its linkage with B7.1 (CD80) or B7.2 (CD86) molecules. CTLA-4 (Cytotoxic T-Lymphocyte Antigen 4) is a CD28 homolog that interacts with B7.1 and B7.2 and, in contrast to CD28, provides an inhibitory signal. However, there are many more molecules that stimulate or inhibit the function of lymphocytes in the immune synapse [18,19].

Certainly, an immunotherapy with the immune checkpoints inhibitors is a breakthrough in the treatment of many cancers. The most important negative immune checkpoints are proteins located on the surface of T lymphocytes: the PD-1 molecule, which regulates T cells activity in peripheral tissues, and the CTLA-4 molecule, which plays the role in regulating lymphocyte functions in lymph nodes during antigen presentation [18,20,21]. It should be noted that understanding the function and regulation of the immune system activity by these molecules has contributed to the huge development of immunotherapy methods, and the discoverers of these molecules—James Allison (for the discovery of the CTLA-4 molecule) and Tasuko Honjo (for the discovery of the PD-1 molecule)—were awarded the Nobel Prize in medicine and physiology in 2018. Ipilimumab (monoclonal antibody anti-CTLA-4), approved for the treatment of metastatic melanoma, represents the first success of immune checkpoints inhibitors therapy [18,20,22].

PD-1 is located on T lymphocytes, NK cells and non-stimulated B lymphocytes, i.e., cells involved in specific immune response [21]. Expression of PD-1 on dendritic cells, macrophages and monocytes may appear after stimulation, e.g., with interferon γ (IFN-γ) during inflammation. In addition, the expression of this molecule may also be enhanced by other pro-inflammatory cytokines inducing PD-1 mRNA transcription in cytotoxic and in helper T lymphocytes [23,24]. The lymphocyte inhibitory signal is transmitted through PD-1 as a result of its interaction with the ligand—the PD-L1 molecule [21,23,24].

PD-L1 molecule is a trans-membrane glycoprotein found mainly on the surface of innate cells (macrophages or monocytes). In healthy people, these cells may show negligible expression of the PD-L1 molecule, whereas, during the ongoing chronic inflammatory process, the expression of this molecule is induced, which is a mechanism protecting against excessive activation of T lymphocytes (PD-L1 interaction with PD-1 extinguishes PD-1-positive cells activity). The abovementioned checkpoint molecules regulate the activity of immune system in physiological conditions. Moreover, PD-L1 expression could also be present on tumor cells and this mechanism allows the tumor cells to escape from the immune system. It should also be remembered that PD-L1 molecule may appear on the surface of tumor cells as early as the appearance of abnormal cells in the body (primary PD-L1 expression on tumor cells) or under the influence of pro-inflammatory cytokines, mainly after IFN-γ stimulation (adaptive expression of PD-L1 on tumor cells) [21,24,25].

A variety of immunomodulatory pathways on lymphocytes, antigen presenting cells and tumor cells have been studied. Tim-3 and LAG-3—members of the immunoglobulin superfamily—have also been shown to play an inhibitory role on lymphocytes. However, GITR, OX40, ICOS, CD137, CD40L and CD27 and other members of the tumor necrosis factor receptor (TNFR) superfamily play costimulatory function on lymphocytes. These inhibitory and stimulatory molecules have been studied as therapeutic targets [26,27].

**Figure 1:**
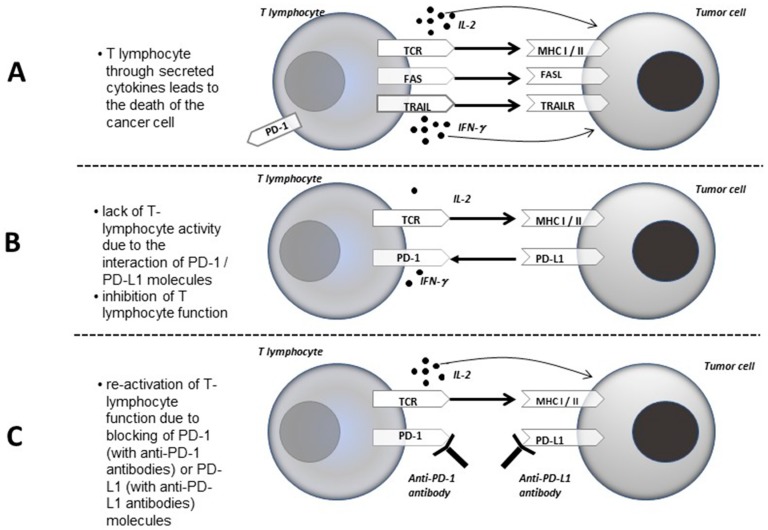
Mechanisms of: killing tumor cells by active T lymphocytes (**A**); blocking their action through the interaction of PD-1 and PD-L1 molecules (**B**); and re-activation of T-cell activity by using anti-PD-1 or anti-PD-L1 antibodies (**C**).

Nowadays, the basic idea of immunotherapy with immune checkpoints inhibitors is to prevent transmission of intracellular inhibitory signal after PD-1 and PD-L1 or CTLA-4 and B7.1/B7.2 interaction. Therefore, the use of monoclonal antibodies which block the PD-1 or PD-L1 molecules is of great interest in cancers treatment (Figure 1). The effectiveness of both groups of antibodies has been proven in many clinical trials, which translated into positive immunotherapeutic registrations in the world [22,28].

## 3. Immune-Check Points Inhibitors—Reality of Lung Cancer Therapy

The treatment protocols for metastatic NSCLC patients, without mutations in epidermal growth factor receptor (*EGFR*) and *BRAF* genes or without rearrangements in anaplastic lymphoma kinase (*ALK*) and *ROS1* genes, were based on chemotherapy, namely platinum doublets in first line setting and docetaxel or pemetrexed in second line treatment. As patients were achieving disease control in about 50% in first line and up to 20–25% in second line therapy, with modest response duration with an overall survival of about 12 months from diagnosis, new treatment strategies leading to improved, durable response rates, translating into OS prolongation without unacceptable toxicities, were an urgent need [29].

### 3.1. PD-1/PD-L1 Checkpoint Inhibitors in Second Line Therapy

The landscape of NSCLC treatment changed in 2015 when CheckMate 017 and CheckMate 057 studies results were published. In the CheckMate 017 phase III trial, 272 patients with locally advanced or metastatic squamous NSCLC, who had disease progression during or after first line chemotherapy, were randomly assigned to receive nivolumab or docetaxel. The median OS was 9.2 months (95% CI: 7.3–13.3) in nivolumab treated group versus 6.0 months (95% CI: 5.1–7.3) in docetaxel treated group (HR = 0.59, 95% CI: 0.44–0.79, *p* < 0.001). The expression of PD-L1 was neither prognostic nor predictive of benefit. CheckMate 057 was conducted based on the same trial design but was dedicated to 582 non-squamous NSCLC patients. The median OS was 12.2 months (95% CI: 9.7–15.0) among 292 patients in the nivolumab group and 9.4 months (95% CI: 8.1–10.7) among 290 patients in the docetaxel group (HR = 0.73, 96% CI: 0.59–0.89, P = 0.002). Nivolumab was associated with longer OS and progression free survival (PFS), and higher objective response rates (ORR) than docetaxel in patients with PD-L1 expression on ≥1%, ≥5%, and ≥10% of tumor cells. Both CheckMate 017 and CheckMate 057 showed lower serious adverse events rate in the nivolumab arms. The CheckMate 017 and CheckMate 057 studies were practice-changing, incorporating nivolumab as a second line treatment standard of care [3,4]. The pooled analysis of the two studies showed superior two-year OS rates (23% for nivolumab vs. 8% for docetaxel in squamous NSCLC patients and 29% with nivolumab vs. 16% with docetaxel in non-squamous NSCLC patients), demonstrating that nivolumab provides a long-term benefit and a favorable tolerability profile compared with docetaxel in this setting [30]. 

The phase II/III KEYNOTE 010 study compared pembrolizumab with docetaxel in second line treatment of locally advanced or advanced NSCLC patients. Pembrolizumab significantly improved the OS, PFS, and ORR of the patients who had PD-L1 expression on ≥1% of tumor cells [1]. Moreover, atezolizumab significantly prolonged OS of previously treated patients with locally advanced or metastatic NSCLC regardless of PD-L1 expression on tumor cells. Pembrolizumab and atezolizumab had a favorable safety profile when compared with docetaxel in KEYNOTE 010 as well as in both phase II (POPLAR) and phase III (OAK) trials [5,31].

According to the results of the abovementioned trials, PD-1 or PD-L1 blockade in second line treatment of NSCLC patients provides better survival and better safety profile than docetaxel, which allowed changing the standard of care for patients progressing after platinum-based first line therapy.

### 3.2. PD-1/PD-L1 Checkpoint Inhibitors in First Line Treatment

The KEYNOTE 024 trial demonstrated that pembrolizumab was better than chemotherapy in patients with advanced NSCLC and with PD-L1 expression on ≥50% of tumor cells. Pembrolizumab led to significantly longer PFS and OS [2]. The more recent KEYNOTE 042 study conducted in patients with PD-L1 expression on ≥1% of tumor cells reported that pembrolizumab was superior to first line chemotherapy with less side effects [32]. 

The KEYNOTE 189 study enrolled patients with advanced nons-quamous NSCLC with no alterations in *EGFR* or *ALK* genes. The study confirmed that the combination of pembrolizumab and chemotherapy resulted in significantly improved PFS and OS in comparison to first line chemotherapy, irrespective of PD-L1 expression [8]. The KEYNOTE 407 phase III study was dedicated to squamous NSCLC, treatment naïve patients and it demonstrated that pembrolizumab combined with chemotherapy is associated with longer median OS (15.9 months in pembrolizumab treated group and 11.3 in chemotherapy alone treated group, HR = 0.64), regardless of PD-L1 expression on tumor cells. Median PFS was 6.4 in patients receiving combined treatment and 4.8 in chemotherapy treated patients (HR = 0.56) with benefit of combined treatment observed across subgroups [33].

In the CheckMate 026 study, nivolumab treatment did not improve OS (median OS 14.4 months for nivolumab vs. 13.2 months for chemotherapy) or PFS (median PFS 4.2 months for nivolumab vs. 5.9 months for chemotherapy) in previously untreated patients with stage IV or recurrence of NSCLC with PD-L1 expression on at least 5% of tumor cells, or in the subgroup of patients with PD-L1 expression on ≥50% of tumor cells. The tumor mutational burden (TMB) was not predictive for benefit of overall survival in nivolumab monotherapy compared to chemotherapy, but a higher ORR (47% vs. 28%) and longer PFS (9.7 vs. 5.8 months) were observed in the high TMB subgroup [11] In CheckMate 227 phase III study, the combination of nivolumab and ipilimumab demonstrated a longer PFS, higher ORR, and comparable adverse events in comparison to chemotherapy in advanced NSCLC with a high TMB (discussed below) [34].

In IMPower 150 clinical trial, advanced NSCLC patients received atezolizumab plus carboplatin plus paclitaxel (ACP), bevacizumab plus carboplatin plus paclitaxel (BCP), or atezolizumab plus BCP (ABCP) The median PFS was longer in the ABCP group than in the BCP group (8.3 vs. 6.8 months, HR = 0.62; 95% CI: 0.52–0.74, P < 0.001); Progression-free survival was also longer in the ABCP group than in the BCP group regardless of *EGFR* or *ALK* genetic alterations, presence of PD-L1 expression on tumor cell, as well as Teff gene-signature expression. Moreover, median OS among the patients without *EGFR* and *ALK* genes alterations was longer in the ABCP group than in the BCP group (19.2 vs. 14.7 months, HR = 0.78, 95% CI: 0.64–0.96; P = 0.02). The safety profile of ABCP was consistent with previously reported safety risks of the individual medicines [10].

According to the published trial results in first line treatment, pembrolizumab monotherapy should be the first choice in patients with PD-L1 expression on at least 50% of tumor cells and no *EGFR* and *ALK* alterations, while for the remaining patients with lower PD-L1 expression pembrolizumab or atezolizumab combined with chemotherapy demonstrated better outcomes than chemotherapy alone. The combination of nivolumab and ipilimumab is beneficial in patients with high TMB, but there are still some limitations (discussed below).

### 3.3. PD-1/PD-L1 Checkpoint Inhibitors as Consolidation after Chemoradiation

PACIFIC was a phase III trial that aimed to compare the efficacy of placebo to durvalumab as consolidation treatment in patients with stage III NSCLC who did not progress after two or more cycles of platinum-based chemoradiotherapy. The study demonstrated that in patients receiving durvalumab the median OS, PFS, and ORR were significantly improved compared to placebo (23.2 vs. 14.6 months, 16.8 vs. 5.6 months and 28.4% vs. 16.0%) [7].

## 4. PD-L1 Expression as an Approved Predictive Factor for Immunotherapy—Shins and Shadows

PD-L1 expression on tumor cells is a confirmed predictive factor for ICI therapy in NSCLC patients investigated in prospective clinical trials. However, the results of clinical trials on the role of PD-L1 expression in qualification for immunotherapy are divergent. This is due to differences in randomization according to different PD-L1 status on tumor and immunological cells [1,2,3,4,5,6,7]. For example, in second line treatment, pembrolizumab was used in patients with any PD-L1 expression, and nivolumab and atezolizumab in patients with or without PD-L1 expression on tumor cells [1,5]. Divergent results of clinical trials on the role of PD-L1 expression as a predictive factor for immunotherapy may result from significant differences in the method of testing and evaluation of PD-L1 expression by immunohistochemistry (IHC). Different monoclonal antibodies clones and reagent kits as well as various equipment for IHC staining were used for the determination of PD-L1 expression on tumor and immune cells in clinical trials with various anti-PD-1 or anti-PD-L1 antibodies (Table 1) [1,2,3,4,5,6,7,8,9,10,11,12]

The results of IHC testing with different antibody clones are not comparable in all NSCLC patients. In addition, the interpretation of the results depends on the pathomorphologist’s experience. Blue Print 1 and 2 studies revealed that four IHC assays (with 22C3, 28-8, SP263, and 73-10 antibody clones) were closely aligned, whereas the fifth IHC assay (with SP142 antibody clone) showed consistently fewer PD-L1 stained tumor cells. All the assays demonstrated PD-L1 expression on immune cells, but with greater variability than on tumor cells. The Blue Print 2 study showed that higher percentage of negative results of PD-L1 expression were obtained in cytoblocks then in histological materials. The authors concluded that the evaluation of PD-L1 expression in cytoblocks has limited reliability. The authors summarized that differences in methods of IHC tests would lead to misclassification of PD-L1 status in some patients [35,36].

Moreover, the expression of PD-L1 in a tumor is known to be very heterogeneous [37]. McLaughlin et al. found a large variation in PD-L1 expression on tumor cells in different parts of the same tumor (from high expression to its complete absence) [38]. Ilie et al. showed 48% of overall discordance rate between results of PD-L1 expression examination on tumor cells in surgically resected tissues and in biopsy specimens [39]. Only 25% of surgical specimens and 74% of biopsy specimens did not express PD-L1. Moreover, PD-L1 expression changes during previous therapies such as radiotherapy or chemotherapy (PD-L1 expression is most often examined in archival materials) [40].

PD-L1 expression on tumor or immune cells is not an ideal predictive factor for immunotherapy for one more reason. Clinical trials showed that the effectiveness of second line treatment with anti-PD-1 or anti-PD-L1 antibodies is possible even in patients without PD-L1 expression on tumor or immune cells. CheckMate 017 study showed that median OS was significantly higher in second line nivolumab therapy compared to docetaxel therapy in squamous cell lung cancer patients regardless of PD-L1 expression on tumor cells [3]. In clinical trial CheckMate 057, the median survival in nivolumab treated patients with PD-L1-negative non-squamous cell lung cancer tumors was similar to the survival of such patients treated with docetaxel [4]. In OAK study, NSCLC patients with undetectable PD-L1 expression on tumor or immune cells also had improved survival with atezolizumab versus docetaxel [5]. On the other hand, a lack of response to immunotherapy is observed in many patients with PD-L1 expression on tumor or immune cells [3,4,5].

These observations lead to the conclusion that it is necessary to search for new predictive factors useful in qualification of NSCLC patients to immunotherapy.

## 5. Beyond PD-L1 Marker—Other Predictive Markers

### 5.1. Tumor Mutation Burden

PD-L1 expression is not a satisfying tool to identify NSCLC patients that might benefit from therapy with immune checkpoints inhibitors. Patients with high PD-L1 expression on ≥50% of tumor cells clearly respond to first line therapy with pembrolizumab, but still some patients with lower or even negative expression of PD-L1 obtain response and benefit of extending progression free survival and overall survival in second line of ICI treatment [4,5,6,7,8,9,10,11,12]. This phenomenon shows that high PD-L1 expression is not the only positive predictive factor for ICI therapy. The Food and Drug Administration (FDA) also approved microsatellite instability (MSI) in tumor cells as an indication for ICI therapy [41].

High tumor mutation burden was presumed to be a potential candidate to become an important predictive factor and tested in this indication in multiple clinical trials. TMB is a quantitative measure of the total number of somatic nonsynonymous mutations per coding area of a tumor genome. TMB potentially translates into a higher neo-antigen load, and a higher chance of effective antigen stimulation that results in an interaction between tumor cells and effector cytotoxic T-cells especially with the ICI presence [42].

Assessment of tumor mutation burden is possible by using next generation sequencing (NGS) technology. There are two most commonly used methods to measure TMB. One of them is whole exome sequencing (WES), which is not feasible for daily clinical practice due to high cost and some limitations resulting in long testing time. Comprehensive genomic profiling (CGP), with two assays approved by the FDA (FoundationOne CDx and MSK-IMPAKT), is more commonly used in clinical trials and potentially in routine practice. Another important issue for TMB is the threshold for positive results, which is not fully consistent across clinical trial results [43,44,45,46,47,48].

Multiple clinical trials and subanalyses addressed the question of the potential role of TMB as a predictive marker in NSCLC patients treated with ICIs. In a retrospective study by Rizvi et al., conducted in 34 advanced NSCLC patients treated with pembrolizumab, high TMB (defined as ≥ 178 nonsynonymous mutations by WES) was associated with better and durable objective responses and benefit in terms of PFS [43].

In 2017, Peters et al. published the results of an exploratory retrospective analysis of a phase III CheckMate 026 study that included 312 NSCLC patients with a PD-L1 expression ≥5% of tumor cells treated with first line chemotherapy or nivolumab. TMB was tested with WES and defined high tertile for >243 mutations and low for <100 mutations [44]. The study showed that high TMB was associated with longer PFS in nivolumab treated patients as compared to chemotherapy (9.7 vs. 5.8 months, HR = 0.62, 95% CI: 0.38–1.0) and higher objective response rate (ORR) (46.8% vs. 28.3%). The study showed that there was no correlation between TMB and PD-L1 expression, also identifying a subgroup of patient of high TMB and high PD-L1 expression that had a higher response rate compared to the subgroup where both factors were low.

CheckMate 012 was a phase I study conducted in 75 advanced NSCLC patients treated with ipilimumab and nivolumab in first line therapy [12]. Patients with high TMB assessed by WES in tumor tissue and in circulating tumor DNA (ct-DNA) showed a higher overall response rate (51% vs. 13%) and a longer progression free survival (HR = 0.41) than patients with low TMB.

In 2018, Ramalingam et al. published the results of the phase II CheckMate 568 study in which 288 stage IV NSCLC systemic treatment-naïve patients were given ipilimumab with nivolumab, regardless of PD-L1 expression. TMB was tested with FoundationOne CDx assay, with four TMB levels of <5, <10, ≥10, and ≥15 mutations/mega base pair (Mut/Mb). High TMB defined as ≥10 Mut/Mb was associated with an overall response rate of 40% [47].

Finally, in the phase III CheckMate 227 study, treatment-naïve NSCLC patients with PD-L1 expression on ≥1% of tumor cells were randomly assigned to treatment with ipilimumab and nivolumab, nivolumab monotherapy or chemotherapy [35]. In patients with PD-L1 expression on ≥1% of tumor cells, treatment arms included ipilimumab with nivolumab, nivolumab with chemotherapy and chemotherapy alone. Hellmann et al. published the results of a part of the study showing the comparison of PFS among patients with a high TMB (defined as ≥10 Mut/Mb) [30]. The study showed that the one-year PFS rate was 42.6% for those treated with ipilimumab and nivolumab, and 13.2% for patients treated with chemotherapy. The median PFS was significantly higher for patients treated with ipilimumab with nivolumab compared to chemotherapy (HR = 0.58, 97.5% CI: 0.41–0.81). The study also showed that the benefit of ipilimumab with nivolumab compared to chemotherapy in patients with high TMB is independent of PD-L1 expression.

The MYSTIC trial results, shown in 2018 as a conference oral presentation, are consistent with CheckMate 227. Although MYSTIC did not meet primary endpoints of OS and PFS improvement in metastatic NSCLC patients with PD-L1 expression on ≥25% of tumor cells treated with durvalumab versus chemotherapy, and durvalumab plus tremelimumab versus chemotherapy, the exploratory analysis in a large dataset showed that high blood TMB (defined as ≥16 Mut/Mb) was associated with better OS for durvalumab with tremelimumab versus chemotherapy with a HR of 0.62 (95% CI: 0.451–0.855) and with a two-year OS of 39% of patients versus 18% [49].

To summarize, TMB seems to become a useful predictive marker for ICI effectiveness in NSCLC patients regardless of the PD-L1 expression, allowing the identification of patients who will benefit from nivolumab plus ipilimumab or durvalumab plus tremelimumab in the subgroup of low or negative PD-L1 expression. However, TMB has important limitations such as long turn-around time of the test, large number of tumors cells required to perform the test, unclear threshold for positive results different across studies and, finally, limited data proving that TMB is predictive in terms of OS in patients treated with ICI combination.

### 5.2. Immunophenotype of Tumor Tissue

TMB and PD-L1 expression are the most validated predictors of benefit from immune checkpoints blockade in advanced NSCLC. However, both mentioned predictive factors are associated with diagnostic issues, which may result in imprecise patients’ qualification to ICI therapy that ends with poor clinical outcome. It seems that other predictive factors should also be considered. Genomic characteristics associated with tumor immune cells infiltration, as well as immunogenic profiling, could have potential role for further advances in treatment selection and patient outcomes after ICI therapy [50,51]. 

In many clinical trials, it is postulated to introduce the analysis of cancerous tissue not only for pathological and molecular diagnosis, but also for the presence of immune cells responsible for anti-tumor immune response. The assessment of selected parameters of the immune system’s activity allows for the prognosis of the disease course, but also provides the possibility of planning the type of effective immunotherapy to increase the activity or modulation of immune system function. The basic tests that should be included in the immunoassay panel are shown in Table 2 [52,53].

Based on the immunological examination of tumor tissue, it is possible to distinguish three basic immune profiles of tumor, which correlate with response to anti-PD-1 and anti-PD-L1 antibodies [15,54]:

1) The immune-inflamed phenotype, so-called “*hot tumor*”, which is characterized by the presence of CD8-positive cytotoxic T lymphocytes as well as CD4-positive memory and regulatory T lymphocytes. Moreover, lymphocyte infiltration is often accompanied by the presence of non-specific response cells. The capacity of immune cells to produce many proinflammatory and effector cytokines could also be detected by mRNA analysis in tumor tissue. Suboptimal or exhausted antitumor immunity is observed in this type of tumors, hence the therapeutic aim is to potentiate the existing immune response.
2) The immune-excluded phenotype, which is characterized by the lack of immune cells invasion into the tumor. The immune cells do not penetrate the tumor core, but instead are retained in the stroma. It is postulated that strongly inhibiting tumor microenvironment produces immunosuppressive metabolic products such as indole 2,3-dioxygenase (IDO) or IL-10 that block the activity of immune cells. Therefore, the therapeutic aim is to reverse tumor immunosuppression and lead the pre-existing antitumor response penetrating into the stroma.
3) The immune-desert phenotype, so-called “*cold tumor*”, which is characterized by the scarcity of T cells either in the tumor core or in the stroma. No immunosuppressive factors are released by tumor or chemokines in the tumor microenvironment. This phenotype probably considers the absence of pre-existing antitumor immunity. Therefore, the therapeutic aim in this type of tumor tissue is concentrated on priming new immune response.

This trichotomy is observed across most solid tumors and its connected with cancer response to immune checkpoints inhibitors therapy. The highest response rate is associated with inflamed phenotype of tumor tissue, but the exact mechanism of action is more complicated. First, the high concentration of proinflammatory cytokines (IFN-γ) stimulates the PD-L1 expression on tumor cells, as well as on non-lymphoid cells. This increases the inhibition of PD-1-positive lymphocytes, which becomes the goal for anti-PD-1 and anti-PD-L1 antibodies [15,54,55]. Additionally, the existing but hampered immune response is strengthened after ICI therapy. The quite opposite situation takes place in “cold tumors”, where there are no inflammatory or lymphoid cells. In this particular situation, the use of ICIs will not have a rational basis. However, PD-L1 expression is not strictly correlated with inflamed phenotype. “Cold tumors” could also express PD-L1. It is well known that higher expression of PD-L1 on tumor cells is associated with a significant clinical response after anti-PD-1 or anti-PD-L1 immunotherapy. However, we have evidence that some PD-L1–negative patients could also respond to this therapy. In many studies conducted on animal models, it has been proven that the presence of PD-L1 molecule is significant not only on tumor cells but, more importantly, on the host immune cells. Tang et al. found that anti–PD-L1 antibodies accumulate in tumor tissues, regardless of the status of PD-L1 expression on tumor cells. PD-L1 on antigen-presenting cells negatively regulated and inhibited T cell function [56]. Finally, PD-L1 blockade inside tumors was not enough to re-activate T cell activity due to impaired T cell trafficking into the tumor. Lin H et al. showed that in PD-L1- or PD-1-negative or immunodeficient mice, PD-L1 blockade, even in tumors with PD-L1 overexpression, had no efficacy [57]. These findings demonstrate that PD-L1 expressed on host immune cells, rather than on tumor cells, plays an essential role in the efficacy of checkpoint blockade therapy. However, some studies indicate that the response to the anti-PD-1 or anti-PD-L1 immunotherapy depends on both factors associated with the tumor itself and with the activity of the host’s immune system [58].

Taken together, the prediction of ICI therapy outcome or precise patient’s selection should be based not only on PD-L1 expression, but on various inflammation signs. Different gene signatures (tumor inflammation signature) have been described to explain the mechanism of cancer responsiveness or resistance to ICIs. The Teff tumor gene expression signature (Teff tGE) is analysis of mRNA expression in tumor tissue for the following genes: *CXCL9*, *INF-γ* and *PD-L1*. The patients with high T effector gene signature compared to those with low T effector gene signature benefited more from atezolizumab therapy than from chemotherapy in POPLAR and IMPower150 studies [15,54,55,59].

It seems that a simpler analysis, which could be performed in any oncological department, is so-called the Lung Immune Prognostic Index (LIPI). The LIPI combines the pre-treatment-derived neutrophil to lymphocyte ratio (dNLR) and lactate dehydrogenase (LDH) level in peripheral blood. The results of a retrospective study conducted by Varga et al. were presented at International Congress on Targeted Anticancer Therapies in Paris in 2019. The authors divided patients with different solid tumors treated with ICIs into three groups according to the following parameters: (1) group with dNLR <3 and normal LDH; (2) group with dNLR >3 or LDH > upper limit of normal; and (3) group with dNLR >3 and LDH >ULN. Survival was correlated with the LIPI stratification. Median OS was 17.8 months in group of patients with low dNLR and normal LDH, compared to 11.68 in the intermediate group and 3.9 months for patients in high dNLR and LDH. The authors suggested that the LIPI calculated prior to immunotherapy initiation could be used to stratify patients into the groups that will benefit from immune checkpoints inhibitors therapy. However, it cannot be ruled out that LIPI is primarily a prognostic and not a predictive factor [60]. 

### 5.3. Microbiome

Human gut microbiome has a rather stable composition at the phylum level. The Gram-negative *Bacteroidetes* are the most numerous bacteria in physiological microbiome of the human gastrointestinal tract (GIT). Gram-positive *Firmicutes* (*Bacilli, Lactobacilli and Diplococci*) are permanently present in GIT of everyone, however the proportions between them can change during the lifetime, depending on the state of health, diet or geography [61,62,63]. *Bacteroidetes* and *Firmicutes* constitute of about 90% of microbiome. *Proteobacteria* and *Actinobacteria* also exist in gut but they are in the minority (10%) [61,62,63,64,65]. It is estimated that human gut microbiome consists of over 1000 different species of bacteria. Functions of bacteria in the digestive tract is to facilitate digestion, to produce vitamins, neurotransmitters (the brain-intestine axis), amino acids, and to compete with pathogenic bacteria [61]. High diversity of gut microbiome leads to promotion of M1 macrophage and Th1 lymphocyte differentiation, helper and cytotoxic T cell activation and upregulation of PD-1 expression on lymphocytes [66].

**Figure 2:**
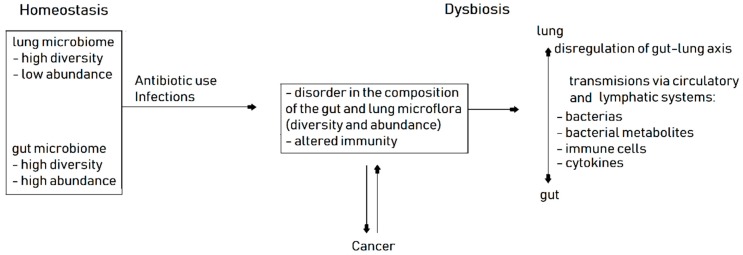
General scheme of microbiome disorders associated with cancer.

Cancer patients’ microbiome could be examined not only in GIT but also in tumor and adjacent tissue [67] (Figure 2). The lung tissue microbiome forms a distinct cluster, largely separated from the microbiome of gut, skin, vagina or oral and nasal cavity. *Proteobacteria* is a dominant phylum (60%) in non-malignant lung tissue surgically resected in NSCLC patients. *Firmicutes*, *Bacteroidetes* and *Actinobacteria* occur in the minority in non-malignant lung tissue. Microbiome diversity increases with the environmental exposure (air pollution and density of population) and with the intensity of tobacco smoking. Number and diversity of bacteria types is significantly higher in non-tumor lung tissues than in tumor tissues and in chronic bronchitis epithelium [68]. Disturbance of lung microbiome composition (dysbiosis) and epithelial disintegration in heavy smokers could be a primary cause of inflammatory process in chronic obstructive pulmonary disease and lung cancer [69]. There is evidence that recurrent antibiotic exposure and resulting dysbiosis also increase lung cancer risk [70].

Nowadays, microbiome has been widely studied in cancer patients as a causative (bacterial metabolites damaging DNA) or immunostimulant agents. Organism immunity depends on the composition of the intestinal microbiome [66]. It seems that in cancer patients gut microbiome could affect patients’ responses to immunotherapy with immune checkpoints inhibitors. Primary resistance to ICIs could be caused by dysbiosis in intestinal microbiome. It has been proven that antibiotics limit ICI efficacy in patients with advanced cancers. Antibiotics reduce gut microbiota diversity and lead to dysbiosis, which may affect effectiveness of ICIs. This was demonstrated in the experiment where anti-PD-1 antibody alone or together with anti-CTLA-4 antibody were used in mice with set MCA-205 sarcoma and RET melanoma. Mice were treated for 14 days with broad-spectrum antibiotic combination (ampicillin, colistin and streptomycin) or were untreated. They were maintained in pathogen-free conditions. Antibiotics significantly decreased the antitumor effects of ICIs resulting in poor response and survival reduction of mice treated with immunotherapy [71].

Derosa et al. examined patients with advanced renal cell carcinoma (RCC) and NSCLC treated with anti-PD-L1 antibody monotherapy or combination immunotherapy. Sixteen patients with RCC and 48 NSCLC patients received antibiotics within 30 days from the beginning of ICI therapy [72]. In RCC patients, antibiotics therapy preceding ICIs was associated with increased risk of early progression (75% versus 22%), decreased median PFS (1.9 versus 7.4 months), and decreased median OS (17.3 versus 30.6 months). In NSCLC patients, the use of antibiotics caused decreased median PFS (1.9 versus 3.8 months) and OS (7.9 versus 24.6 months). The authors concluded that modulation of antibiotics-related dysbiosis and gut microbiota composition may be a strategy to improve clinical outcomes with ICIs.

Routy et al. examined the impact of antibiotic therapy in patients with advanced NSCLC (n = 140), RCC (n = 67), or urothelial carcinoma (n = 42) who received anti-PD-1 or anti-PD-L1 antibodies after one or several prior therapies [68]. Out of 249 patients, 69 (28%) were prescribed antibiotics (β-lactams, fluoroquinolones, or macrolides) within two months before, or one month after the first administration of ICIs. PFS and OS were significantly shorter in the antibiotics-treated patients regardless of the type of cancer. In univariate and multivariate Cox regression analyses, the use of antibiotics represented a predictor of resistance to PD-1 or PD-L1 blockade, independent from classical prognostic markers in NSCLC and RCC patients. A validation cohort of 239 advanced NSCLC patients confirmed the negative impact of antibiotics therapy on survival of patients treated with anti-PD-1 or anti-PD-L1 antibodies.

For the first time, the relationship between the occurrence of specific bacterial species in gut microbiome and the effectiveness of anti-CTLA-4 or anti-PD-1/PD-L1 antibodies have been observed in melanoma patients. Vetizou et al. found that tumors in antibiotics-treated or germ-free mice did not respond to CTLA-4 blockade [73]. This effect was overcome by gavage with *Bacteroides fragilis*, by immunization with polysaccharides from this bacterium or by adoptive transfer of *B. fragilis*-specific T cells. Fecal microbiota transplantation (FMT) from humans to mice confirmed that treatment of melanoma patients with ipilimumab favored the outgrowth of *B. fragilis* with anticancer properties. In the same year, Sivan et al. postulated that commensal *Bifidobacterium* promotes anti-PD-L1 treatment efficacy via the antitumor T cells recruiting and activation, interferon and proinflammatory cytokine production and maturation of dendritic cells, which reinforce the presentation of neoantigens [74]. They used mouse xenografts model with subcutaneous administration of melanoma cells. Oral administration of *Bifidobacterium* (cocktail of *B. bifidum*, *B. longum*, *B. lactis*, and *B. breve*) to the xenografts improved tumor control by PD-L1 antibody. Gopalakrishnan et al. indicated that high abundance of *Faecalibacterium* and *Clostridales* (especially *Ruminococcaceae* family) belonging to the *Firmicutes,* as well as high diversity of gut microbiome, elevated response rate in patients receiving anti-PD-1 treatment [75]. Matson et al. showed that *Bifidobacterium longum*, *Collinsella aerofaciens*, and *Enterococcus faecium* were more abundant in patients with response to anti-PD-L1 antibody [76]. Reconstitution of germ-free mice with fecal material from responding patients lead to augmented T cell response through increase neoantigen specific cytotoxic T cells and decrease regulatory T cells in tumors.

The findings regarding the role of microbiome in the efficacy of cancer immunotherapy fit in the Human Microbiome Project implemented by National Institute of Health. The initial phase of the project, established in 2008, characterized the microbial communities from 300 healthy individuals, across several different sites of the human body: nasal and oral cavity, skin, gastrointestinal tract and urogenital tract. Sequencing of prokaryotic genes for subunit 16 of ribosomal RNA (16S rRNA) with nine hypervariable regions (V1–V9) was used to characterize the human microbiome. Highly conserved fragments are located between these hypervariable regions, which allows designing universal primers for distinguishing of different bacterial taxa. Metagenomic whole-genome shotgun sequencing on next generation sequencing platforms (NGS) provided information about the composition of the microbiota at the gene count or metagenomic species (MGS) levels. Specific bioinformatics tools have been developed for analysis of 16S rRNA gene sequence by its comparison with known bacterial sequences from database. All unknown sequences were taken into account; however, they were described as unclassified bacteria. Traditional microbiological methods do not allow defining taxonomic assignment, abundance and diversity of whole microbiome and they cannot longer be used for this purpose [77].

Routly et al. made metagenomics analysis of gut microbiota of 60 NSCLC and 40 RCC patients. They isolated stool total DNA from patients at diagnosis, before starting therapy and sequentially after PD-1 blockade. Analysis showed that the higher gene count or MGS correlated positively with the clinical response defined by the absence of progression of disease at six months after initiation of ICIs [72].

After profiling microbiome samples from NSCLC and RCC patients, Routy et al. found that patients who respond to ICIs are characterized by overrepresentation of unclassified and classified *Firmicutes* and distinct bacterial genera such as *Akkermansia* and *Alistipes* [72]. In particular, the large proportion of *Akkermansia muciniphila* in the microbiome was conducive to the long-term responses to ICI therapy. The authors also observed a higher incidence of *Enterococcus hirae* in NSCLC patients with response to ICI therapy. FMT from cancer patients, who responded to ICIs, into antibiotic-treated or germ-free mice, improved the antitumor effects of PD-1 blockade, while FMT from nonresponding patients did not improve response to immunotherapy. Routy et al. also analyzed the immunological changes in lymph nodes and in tumors elicited by oral gavage with a combination of *Accermansia muciniphila* and *Enteroccocus hirae*, as well as anti-PD-1 antibody injection. In mouse model, T helper cells acquired expression of chemokine receptor CCR9 and Th1-associated chemokine receptor CXCR3, both in lymph nodes and in tumors. Immunohistochemical studies revealed the formation of intratumoral granulomas and increased presence of regulatory T cells (Foxp3-positive cells). Dendritic cells secreted more IL-12, which is involved in the immunogenicity of PD-1 blockade in eubiotic conditions.

Botticelli at al. studied gut microbiome in NSCLC patients treated with nivolumab and in healthy people [78]. Metagenomics analysis using NGS technique showed higher level of *Rikenellaceae*, *Prevotella*, *Streptococcus*, *Lactobacillus*, *Bacteroides plebeius*, *Oscillospira* and *Enterobacteriaceae* in NSCLC patients than in healthy controls. In NSCLC patients with response to nivolumab therapy, *Ruminococcus bromii*, *Dialister* and *Sutterella* were less abundant and increased levels of *Akkermansia muciniphila*, *Bifidobacterium longum*, *Faecalibacterium prausnitzi*, *Propionibacterium acnes*, *Veillonella parvula*, *Staphylococcus aureus* and *Peptostreptococcus* were observed. Presence of *Clostridium perfringens* was reduced during nivolumab treatment. This study shows that the original composition of the gut microbiome may influence the response to immunotherapy, however immunotherapy also affects the composition of intestinal microbiome. ICIs could participate in recruitment of bacteria that assist in the anti-cancer response in a not completely understood mechanism.

## 6. Summary

PD-L1 expression on tumor cells and tumor mutation burden are the only molecular predictive factors used in the qualification of NSCLC patients to the first line immunotherapy. However, no molecular predictive factors are required in the qualification for first line combined treatment with chemotherapy and immunotherapy and for second line monotherapy with anti-PD-1 or anti-PD-L1 antibodies. Indeed, the effectiveness of immunotherapy could be observed in patients without PD-L1 expression on tumor cells or with low TMB. Moreover, sometimes no response to ICI therapy occurs despite the predisposition to the treatment. The methods of PD-L1 expression assessment and TMB testing have not been decisively defined yet, hence it is necessary to search for new predictive factors for ICI therapy. The presence of inflammatory cells infiltrating the tumor and the ability of lymphocytes to produce pro-inflammatory factors may increase the effectiveness of immunotherapy. Moreover, the immunophenotype of tumors may suggest the necessity of the use of different immunotherapy methods combinations. Finally, the diversity of microbiome and the presence of certain bacterial species (e.g., *Accermansia muciniphila*) may increase the efficacy of immunotherapy. It is possible that microbiome reconstitution in cancer patients with dysbiosis (e.g., after antibiotics therapy) may sensitize them to immunotherapy. All the above indicates the necessity of powerful personalized diagnostic and therapeutic approach, including a comprehensive assessment of PD-L1, TMB and microbiome profiling. This approach may lead to implementation of the most effective treatment, with particular attention to immunotherapy, with relatively low side effects for patients.

## Figures and Tables

**Table 1:** Comparison of the method of IHC testing used to evaluate the expression of PD-L1 on cancer and immune cells in NSCLC patients.

Immune-Check Points Inhibitors	Target	Anti-PD-L1 Monoclonal Antibody Clone in IHC Method	Epitope for Anti-PD-L1 Antibody Binding	IHC Platforms	Assessment Methods
Nivolumab (CheckMate 057 and 017)	PD-1	28-8	Extracellular	Dako Link 48	Tumor cells <1% vs. ≥1% <5% vs. ≥5% <10% vs. ≥10%
Pembrolizumab (KEYNOTE 010)	PD-1	22C3	Extracellular		Tumor cells ≥1% ≥50%
Atezolizumab (OAK)	PD-L1	SP142	Cytoplasmic	Ventana Benchmark	Tumor cells <1% ≥1% to <5% ≥5% to <10% ≥10% to <50% ≥50% Immune cells <1% ≥1% to <5% ≥5% to <10% ≥10%
Durvalumab (PACIFIC, MYSTIC)	PD-L1	SP263	Cytoplasmic		Tumor cells <25% ≥25%

**Table 2:** The basic immunological parameters that can be analyzed in tumor tissue.

Infiltration location	Type of Cells Infiltrating Tumor Tissue	Density of Cells Infiltrating Tumor Tissue	Functional Characteristic of Cells Infiltrating Tumor Tissue
tumor core marginal infiltration lack of any infiltration	cytotoxic T lymphocytes (CD3+CD8+) memory T lymphocytes (CD45RO+) regulatory T lymphocytes (CD4+, CD25+, FoxP3+) macrophages of M1 or M2 type dendritic cells	number of immune cells evaluated in mm^2^ tumor tissue	cytokine production (IFN-γ, IL-10, IL-12) in the tumor environment and its expression in immune cells the presence of cytotoxic granules (granzymes A and B, perforyne, granulisin) chemokine production and expression of receptors for them

## References

[1] Herbst R.S., Baas P., Kim D.W., Felip E., Pérez-Gracia J.L., Han J.Y., Molina J., Kim J.H., Arvis C.D., Ahn M.J. (2016). Pembrolizumab versus docetaxel for previously treated, PD-L1-positive, advanced non-small-cell lung cancer (KEYNOTE-010): A randomised controlled trial. Lancet.

[2] Reck M., Abrau D.R., Robinson A.G., Robinson A.G., Hui R., Csőszi T., Fülöp A., Gottfried M., Peled N., Tafreshi A. (2016). Pembrolizumab versus chemotherapy for PD-L1–positive non-small-cell lung cancer. N. Engl. J. Med..

[3] Brahmer J., Karen L., Reckamp M.D., Crinò L., Eberhardt W.E., Poddubskaya E., Antonia S., Pluzanski A., Vokes E.E., Holgado E. (2015). Nivolumab versus docetaxel in advanced squamous-cell non-small-cell lung cancer. N. Engl. J. Med..

[4] Borghaei H., Paz-Ares L., Horn L., Spigel D.R., Steins M., Ready N.E., Chow L.Q., Vokes E.E., Felip E., Holgado E. (2015). Nivolumab versus docetaxel in advanced nonsquamous non-small-cell lung cancer. N. Engl. J. Med..

[5] Rittmeyer A., Barlesi F., Waterkamp D., Park K., Ciardiello F., von Pawel J., Gadgeel S.M., Hida T., Kowalski D.M., Dols M.C. (2017). Atezolizumab versus docetaxel in patients with previously treated non-small-cell lung cancer (OAK): A phase 3, open-label, multicentre randomised controlled trial. Lancet.

[6] Rizvi N.A., Brahmer J.R., Ou S.-H.I., Segal N.H., Khleif S., Gutierrez W.J.H.M., Schoffski P., Hamid O., Weiss J., Lutzky J. (2015). Safety and clinical activity of MEDI4736, an anti-programmed cell death-ligand 1 (PD-L1) antibody, in patients with non-small cell lung cancer (NSCLC). J. Clin. Oncol..

[7] Antonia S.J., Villegas A., Daniel D., Vicente D., Murakami S., Hui R., Yokoi T., Chiappori A., Lee K.H., de Wit M. (2017). Durvalumab after chemoradiotherapy in stage III non–small-cell lung cancer. N. Engl. J. Med..

[8] Gandhi L., Rodríguez-Abreu D., Gadgeel S., Esteban E., Felip E., De Angelis F., Dómine M., Clingan P., Hochmair M.J., Powell S.F. (2018). Pembrolizumab plus chemotherapy in metastatic non–small-cell lung cancer. N. Engl. J. Med..

[9] Paz-Ares L., Luft A., Vicente D., Tafreshi A., Gümüş M., Mazières J., Hermes B., Şenler F.Ç., Csőszi T., Fülöp A. (2018). Pembrolizumab plus chemotherapy for squamous non–small-cell lung cancer. N. Engl. J. Med..

[10] Socinski M.A., Jotte R.M., Cappuzzo F., Orlandi F., Stroyakovskiy D., Nogami N., Rodríguez-Abreu D., Moro-Sibilot D., Thomas C.A., Barlesi F. (2018). Atezolizumab for first-line treatment of metastatic non-squamous NSCLC. N. Engl. J. Med..

[11] Carbone D.P., Reck M., Paz-Ares L., Creelan B., Horn L., Steins M., Felip F., van den Heuvel M.M., Ciuleanu T.E., Badin F. (2017). First-Line Nivolumab in Stage IV or Recurrent Non-Small-Cell Lung Cancer. N. Engl. J. Med..

[12] Hellmann M.D., Ciuleanu T.E., Pluzanski A., Lee J.S., Otterson G.A., Audigier-Valette C., Minenza E., Linardou H., Burgers S., Salman P. (2018). Nivolumab plus Ipilimumab in Lung Cancer with a High Tumor Mutational Burden. N. Engl. J. Med..

[13] Kowalski D.M., Reinmuth N., Orlov S.V., Fischer J.R., Sugawara S., Mandziuk S., Medine D.M., Novello S., Takeda Y., Soo R.A. (2018). ARCTIC: Durvalumab + tremelimumab and durvalumab monotherapy vs. SoC in 3L advanced NSCLC treatment. Ann Oncol..

[14] Barlesi F., Vansteenkiste J., Spigel D., Ishii H., Garassino M., de Marinis F., Özgüroğlu M., Szczesna A., Polychronis A., Uslu R. (2018). Avelumab versus docetaxel in patients with platinum-treated advanced non-small-cell lung cancer (JAVELIN Lung 200): An open-label, randomised, phase 3 study. Lancet Oncol..

[15] Brahmer J.R., Lacchetti C., Schneider B.J., Atkins M.B., Brassil K.J., Caterino J.M., Chau I., Ernstoff M.S., Gardner J.M., Ginex P. (2018). National Comprehensive Cancer Network. Management of Immune-Related Adverse Events in Patients Treated with Immune Checkpoint Inhibitor Therapy: American Society of Clinical Oncology Clinical Practice Guideline. J. Clin. Oncol..

[16] Fuentes-Antrás J., Provencio M., Díaz-Rubio E. (2018). Hyperprogression as a distinct outcome after immunotherapy. Cancer Treat. Rev..

[17] Forde P.M., Chaft J.E., Smith K.N., Anagnostou V., Cottrell T.R., Hellmann M.D., Zahurak M., Yang S.C., Jones D.R., Broderick S. (2018). Neoad-juvant PD-1 blockade in resectable lung cancer. N. Engl. J. Med..

[18] Chen D.S., Mellman I. (2013). Oncology meets immunology: The cancer-immunity cycle. Immunity.

[19] Chen D.S., Mellman I. (2017). Elements of cancer immunity and the cancer-immune set point. Nature.

[20] Ott P.A., Hodi F.S., Robert C. (2013). CTLA-4 and PD-1/PD-L1 blockade: New immunotherapeutic modalities with durable clinical benefit in melanoma patients. Clin. Cancer Res..

[21] Dong Y., Sun Q., Zhang X. (2017). PD-1 and its ligands are important immune checkpoints in cancer. Oncotarget.

[22] Bagley S.J., Bauml J.M., Langer C.J. (2015). PD-1/PD-L1 immune checkpoint blockade in non-small cell lung cancer. Clin. Adv. Hematol. Oncol..

[23] Riella L.V., Paterson A.M., Sharpe A.H., Chandraker A. (2012). Role of the PD-1 pathway in the immune response. Am. J. Transplant..

[24] Tumeh P.C., Harview C.L., Yearley J.H., Shintaku I.P., Taylor E.J., Robert L., Chmielowski B., Spasic M., Henry G., Ciobanu V. (2014). PD-1 blockade induces responses by inhibiting adaptive immune resistance. Nature.

[25] Kataoka K., Ogawa S. (2016). Genetic biomarkers for PD-1/PD-L1 blockade therapy. Oncoscience.

[26] Mahoney K.M., Rennert P.D., Freeman G.J. (2015). Combination cancer immunotherapy and new immunomodulatory targets. Nat Rev Drug Discov..

[27] Moon E.K., Langer C.J., Albelda S.M. (2017). The era of checkpoint blockade in lung cancer: Taking the brakes off the immune system. Ann. Am. Thorac. Soc..

[28] Iwai Y., Hamanishi J., Chamoto K., Honjo T. (2017). Cancer immunotherapies targeting the PD-1 signaling pathway. J. Biomed. Sci..

[29] Shepherd F.A., Pereira J.R., Ciuleanu T., Ciuleanu T., Tan E.H., Hirsh V., Thongprasert S., Campos D., Maoleekoonpiroj S., Smylie M. (2005). Erlotinib in previously treated non–small-cell lung cancer. N. Engl. J. Med..

[30] Horn L., Spigel D.R., Vokes E.E., Holgado E., Ready N., Steins M., Poddubskaya E., Borghaei H., Felip E., Paz-Ares L. (2017). Nivolumab versus docetaxel in previously treated patients with advanced non-small-cell lung cancer: Two-year outcomes from two randomized, open-label, phase III trials (CheckMate 017 and CheckMate 057). J. Clin. Oncol..

[31] Fehrenbacher L., Spira A., Ballinger M., Kowanetz M., Vansteenkiste J., Mazieres J., Park K., Smith D., Artal-Cortes A., Lewanski C. (2016). Atezolizumab versus docetaxel for patients with previously treated non-small-cell lung cancer (POPLAR): A multicentre, open-label, phase 2 randomised controlled trial. Lancet.

[32] Lopes G., Wu Y.L., Kudaba I., Kowalski D., Cho B.C., Castro G., Srimuninnimit V., Bondarenko I., Kubota K., Lubiniecki G.M. (2018). Pembrolizu- mab (pembro) versus platinum-based chemo- therapy (chemo) as first-line therapy for advanced/metastatic NSCLC with a PD-L1 tumor proportion score (TPS) ≥1%: Open-label, phase 3 KEYNOTE-042 study. J. Clin. Oncol..

[33] Paz-Ares L.G., Luft A., Tafreshi A., Gumus M., Mazieres J., Hermes B., Senler F.C., Fülöp A., Rodriguez-Cid J., Sugawara S. (2018). Phase 3 study of carboplatin-paclitaxel/nab-paclitaxel (chemo) with or without pembrolizumab (pem- bro) for patients (pts) with metastatic squamous (sq) non-small cell lung cancer (NSCLC). J. Clin. Oncol..

[34] Hellmann M.D., Nathanson T., Rizvi H., Creelan B.C., Sanchez-Vega F., Ahuja A., Ni A., Novik J.B., Mangarin L.M.B., Abu-Akeel M. (2018). Genomic 19. Features of Response to Combination Immunotherapy in Patients with Advanced Non-Small-Cell Lung Cancer. 20. Cancer Cell.

[35] Hirsch F.R., McElhinny A., Stanforth D., Ranger-Moore J., Jansson M., Kulangara K., Richardson W., Towne P., Hanks D., Vennapusa B. (2016). PD-L1 immunohistochemistry assays for lung cancer: Results from phase 1 of the Blueprint PD-L1 IHC assay comparison project. J. Thorac. Oncol..

[36] Tsao M.S., Kerr K.M., Kockx M., Beasley M.B., Borczuk A.C., Botling J., Bubendorf L., Chirieac L., Chen G., Chou T.Y. (2018). PD-L1 immunohistochemistry comparability study in real-life clinical samples: Results of Blueprint Phase 2 Project. J. Thorac. Oncol..

[37] Munari E., Zamboni G., Marconi M., Sommaggio M., Brunelli M., Martignoni G., Netto G.J., Moretta F., Mingari M.C., Salgarello M. (2017). PD-L1 expression heterogeneity in non-small cell lung cancer: Evaluation of small biopsies reliability. Oncotarget.

[38] McLaughlin J., Han G., Schalper K.A., Carvajal-Hausdorf D., Pelekanou V., Rehman J., Velcheti V., Herbst R., LoRusso P., Rimm D.L. (2016). Quantitative assessment of the heterogeneity of PD-L1expression in non-small-cell lung cancer. JAMA Oncol..

[39] Ilie M., Long-Mira E., Bence C., Butori C., Lassalle S., Bouhlel L., Fazzalari L., Zahaf K., Lalvée S., Washetine K. (2016). Comparative study of the PD-L1 status between surgically resected specimens and matched biopsies of NSCLC patients reveal major discordances: A potential issue for anti-PD-L1 therapeutic strategies. Ann. Oncol..

[40] Sheng J., Fang W., Yu J., Chen N., Zhan J., Ma Y., Yang Y., Huang Y., Zhao H., Zhang L. (2016). Expression of programmed death ligand-1 on tumor cells varies pre and post chemotherapy in non-small cell lung cancer. Sci. Rep..

[41] Chang L., Chang M., Chang H.M., Chang F. (2018). Microsatellite Instability: A Predictive Biomarker for Cancer Immunotherapy. Appl. Immunohistochem. Mol. Morphol..

[42] Boyiadzis M.M., Kirkwood J.M., Marshall J.L., Pritchard C.C., Azad N.S., Gulley J.L. (2018). Significance and implications of FDA approval of pembrolizumab for biomarker-defined disease. J. Immunother. Cancer.

[43] Rizvi N.A., Hellmann M.D., Snyder A., Kvistborg P., Makarov V., Havel J.J., Lee W., Yuan J., Wong P., Ho T.S. (2015). Cancer immunology. Mutational landscape determines sensitivity 17. to PD-1 blockade in non-small cell lung cancer. Science.

[44] Peters S., Creelan B.D., Hellmann M., Socinski M.A., Reck M., Bhagavatheeswaran P., Chang H., Geese W.J., Paz-Ares L., Carbone D.P. (2017). Abstract CT082: Impact of tumor mutation burden on the efficacy of first-line nivolumab in stage iv or recurrent non-small 18. cell lung cancer: An exploratory analysis of CheckMate 026. Cancer Res..

[45] Rizvi H., Sanchez-Vega F., La K., Chatila W., Jonsson P., Halpenny D., Plodkowski A., Long N., Sauter J.L., Rekhtman N. (2018). Molecular Determinants of Response to Anti-Programmed Cell Death (PD)-1 and Anti-Programmed Death-Ligand 1 (PD-L1) Blockade in Patients with Non-Small-Cell Lung Cancer Profiled with Targeted Next-Generation Sequencing. J. Clin. Oncol..

[46] Gandara D.R., Paul S.M., Kowanetz M., Schleifman E., Zou W., Li Y., Rittmeyer A., Fehrenbacher L., Otto G., Malboeuf C. (2018). Blood-based tumor mutational burden as a predictor of clinical benefit in non-small-cell lung cancer patients treated with atezolizumab. Nat. Med..

[47] Ramalingam S.S., Hellmann M.D., Awad M.M., Borghaei H., Gainor J., Brahmer J., Spigel D.R., Reck M., O’Byrne K.J., Paz-Ares L. (2018). Tumor mutational burden (TMB) as a biomarker for clinical benefit from dual checkpoint blockade with nivolumab (nivo) + ipilimumab (ipi) in first-line (1L) non-small cell lung cancer (NSCLC): Identification of TMB cutoff from CheckMate 568. Cancer Res..

[48] Hellmann M.D., Callahan M.K., Awad M.M., Calvo E., Ascierto P.A., Atmaca A., Rizvi N.A., Hirsch F.R., Selvaggi G., Szustakowski J.D. (2018). Tumor Mutational Burden and Efficacy of Nivolumab Monotherapy and in Combination with Ipilimumab in Small-Cell Lung Cancer. Cancer Cell.

[49] Rizvi N., Cho B.C., Reinmuth N., Lee K.H., Ahn M., Luft A., van den Heuvel M., Cobo M., Smolin A., Vicente D. (2018). Durvalumab with or without tremelimumab vs. platinum-based chemotherapy as first-line treatment for metastatic non-small cell lung cancer: MYSTIC. ESMO Congress (ESMO 2018) 19–23 October 2018, Munich, Germany. Ann. Oncol..

[50] Goodman A.M., Kato S., Bazhenova L., Patel S.P., Frampton G.M., Miller V., Stephens P.J., Daniels G.A., Kurzrock R. (2017). Tumor mutational burden as an independent predictor of response to immunotherapy in diverse cancers. Mol. Cancer Ther..

[51] Gadgeel S.M. (2016). Personalized therapy of non-small cell lung cancer (NSCLC). Adv. Exp. Med. Biol..

[52] Galon J., Mlecnik B., Bindea G., Angell H.K., Berger A., Lagorce C., Lugli A., Zlobec I., Hartmann A., Bifulco C. (2014). Towards the introduction of the ‘Immunoscore’ in the classification of malignant tumours. J. Pathol..

[53] Kirilovsky A., Marliot F., El Sissy C., Haicheur N., Galon J., Pagès F. (2016). Rational bases for the use of the Immunoscore in routine clinical settings as a prognostic and predictive biomarker in cancer patients. Int. Immunol..

[54] Fridman W.H., Zitvogel L., Sautès-Fridman C., Kroemer G. (2017). The immune contexture in cancer prognosis and treatment. Nat. Rev. Clin. Oncol..

[55] Karachaliou M., Gonzalez-Cao M., Crespo G., Drozdowskyj A., Aldeguer E., Gimenez-Capitan A., Teixido C., Molina-Vila M.A., Viteri S., De Los Llanos Gil M. (2018). Interferon gamma, an important marker of response to immune checkpoint blockade in non-small cell lung cancer and melanoma patients. Ther. Adv. Med. Oncol..

[56] Tang H., Liang Y., Anders R.A., Taube J.M., Qiu X., Mulgaonkar A., Liu X., Harrington S.M. (2018). PD-L1 on host cells is essential for PD-L1 blockade–mediated tumor regression. J. Clin. Investig..

[57] Lin H., Wei S., Hurt E.M., Green M.D., Zhao L., Vatan L., Szeliga W., Herbst R., Harms P.W., Fecher L.A., Vats P. (2018). Host expression of PD-L1 determines efficacy of PD-L1 pathway blockade–mediated tumor regression. J. Clin. Investig..

[58] Li H., McSharry M., Bullock B., Nguyen T.T., Kwak J., Poczobutt J.M., Sippel T.R., Heasley L.E., Weiser-Evans M.C., Clambey E.T. (2017). The tumor microenvironment regulates sensitivity of murine lung tumors to PD-1/PD-L1 antibody blockade. Cancer Immunol. Res..

[59] Herbst R.S., Soria J.C., Kowanetz M., Fine G.D., Hamid O., Gordon M.S., Sosman J.A., McDermott D.F., Powderly J.D., Gettinger S.N. (2014). Predictive correlates of response to the anti-PD-L1 antibody MPDL3280A in cancer patients. Nature.

[60] Varga A., Bernard-Tessier A., Auclin E., Mezquita Pérez L., Baldini C., Planchard D., Marabelle A., Hollebecque A., Besse B., Massard C. (2019). Applicability of the Lung Immune Prognostic Index (LIPI) in patients with metastatic solid tumors when treated with Immune Checkpoint Inhibitors (ICI) in early clinical trials. Ann Oncol..

[61] Lozupone C.A., Stombaugh J.I., Gordon J.I., Jansson J.K., Knight R. (2012). Diversity, stability and resilience of the human gut microbiota. Nature.

[62] Sekirov I., Russell S.L., Antunes L.C., Finlay B.B. (2010). Gut microbiota in health and disease. Physiol. Rev..

[63] Kim S., Covington A., Pamer E.G. (2017). The intestinal microbiota: Antibiotics, colonization resistance, and enteric pathogens. Immunol. Rev..

[64] Tap J., Mondot S., Levenez F., Pelletier E., Caron C., Furet J.P., Ugarte E., Muñoz-Tamayo R., Paslier D.L., Nalin R. (2009). Towards the human intestinal microbiota phylogenetic core. Environ. Microbiol..

[65] Federici E., Prete R., Lazzi C., Pellegrini N., Moretti M., Corsetti A., Cenci G. (2017). Bacterial Composition, Genotoxicity, and Cytotoxicity of Fecal Samples from Individuals Consuming Omnivorous or Vegetarian Diets. Front. Microbiol..

[66] Round J.L., Mazmanian S.K. (2009). The gut microbiota shapes intestinal immune responses during health and disease. Nat. Rev. Immunol..

[67] Cummins J., Tangney M. (2013). Bacteria and tumours: Causative agents or opportunistic inhabitants?. Infect. Agent Cancer.

[68] Yu G., Gail M.H., Consonni D., Carugno M., Humphrys M., Pesatori A.C., Caporaso N.E., Goedert J.J., Ravel J., Landi M.T. (2016). Characterizing human lung tissue microbiota and its relationship to epidemiological and clinical features. Genome Biol..

[69] Erb-Downward J.R., Thompson D.L., Han M.K., Freeman C.M., McCloskey L., Schmidt L.A., Young V.B., Toews G.B., Curtis J.L., Sundaram B. (2011). Analysis of the lung microbiome in the “healthy” smoker and in COPD. PLoS ONE.

[70] Boursi B., Mamtani R., Haynes K., Yang Y.X. (2015). Recurrent antibiotic exposure may promote cancer formation—Another step in understanding the role of the human microbiota?. Eur. J. Cancer.

[71] Routy B., Le Chatelier E., Derosa L., Duong C.P.M., Alou M.T., Daillère R., Fluckiger A., Messaoudene M., Rauber C., Roberti M.P. (2018). Gut microbiome influences efficacy of PD-1-based immunotherapy against epithelial tumors. Science.

[72] Derosa L., Hellmann M.D., Spaziano M., Halpenny D., Fidelle M., Rizvi H., Long N., Plodkowski A.J., Arbour K.C., Chaft J.E. (2018). Negative association of antibiotics on clinical activity of immune checkpoint inhibitors in patients with advanced renal cell and non-small-cell lung cancer. Ann. Oncol..

[73] Vétizou M., Pitt J.M., Daillère R., Lepage P., Waldschmitt N., Flament C., Rusakiewicz S., Routy B., Roberti M.P., Duong C.P. (2015). Anticancer immunotherapy by CTLA-4 blockade relies on the gut microbiota. Science.

[74] Sivan A., Corrales L., Hubert N., Williams J.B., Aquino-Michaels K., Earley Z.M., Benyamin F.W., Lei Y.M., Jabri B., Alegre M.L. (2015). Commensal Bifidobacterium promotes antitumor immunity and facilitates anti-PD-L1 efficacy. Science.

[75] Gopalakrishnan V., Spencer C.N., Nezi L., Reuben A., Andrews M.C., Karpinets T.V., Prieto P.A., Vicente D., Hoffman K., Wei S.C. (2018). Gut microbiome modulates response to anti-PD-1 immunotherapy in melanoma patients. Science.

[76] Matson V., Fessler J., Bao R., Chongsuwat T., Zha Y., Alegre M.L., Luke J.J., Gajewski T.F. (2018). The commensal microbiome is associated with anti-PD-1 efficacy in metastatic melanoma patients. Science.

[77] Lloyd-Price J., Mahurkar A., Rahnavard G., Crabtree J., Orvis J., Hall A.B., Brady A., Creasy H.H., McCracken C., Giglio M.G. (2017). Strains, functions and dynamics in the expanded Human Microbiome Project. Nature.

[78] Botticelli A., Putignani L., Zizzari I., Del Chierico F., Reddel S., DI Pietro F., Quagliarello A., Onesti C.E., Raffaele G., Mazzuca F. (2018). Changes of microbiome profile during nivolumab treatment in NSCLC patients. J. Clin. Oncol..

